# Supplementary material for: Overview of Transcriptomic Research on Type 2 Diabetes: Challenges and Perspectives
Source: Genes (Basel). 2022 Jun 30;13(7):1176. doi: 10.3390/genes13071176 (PMC9319211; doi:10.3390/genes13071176)
Supplement: Supplementary file 1 [file genes-13-01176-s001.zip › genes-1784671-supplementary.pdf]

Table S1 The main DEGs and biological processes identified by transcriptome studies

**Differentially expressed genes**	**Biological functions / enriched pathways**	**References**
EGR2, PTGS2, IL1B	Immune response	[16]
ILF2, IL1RL1, IL4R, IL9R, LILRB2, LILRB4, MAPK11, GPX1, TNFRSF13B	T cell signaling and activation, nucleic acid metabolic process, signal transduction	[17]
IRX5, THC2540257, THC2521898, WDR49, RNF150, HLA-DQA1, MED18, THC2667190, GNL3L, CENPI, OLFM4, ENST00000382691, CEACAM8, WIF1, H,LA-DQA2	Innate and adaptive immunity, primary immunodeficiency signaling, interferon signaling, complement system, inositol metabolism, Wnt/β-catenin signaling, LXR/RXR activation, tight junction signaling, cleavage and polyadenyation of pre-mRNA	[18]
UBE2A, UBE2B, UBE2E1, UBE2E3, UBE2G1, MARCH8, ANAPC10, ANAPC13, ANAPC5, FBXO11, COPS2, COPS3, PSMD11, PSMD12, USP1	Glucose metabolism, ion channels, exocytosis, insulin and insulin signaling, apoptosis, oxidative stress, regeneration, cell cycle, ubiquitin–proteasome system	[20]
EGF, FAM46C, HBEGF, ID1, SH3BGRL2, TMEM158, VEPH1	Immune response, lipid metabolism, transcription, apoptosis, oxidative stress, RAS pathway	[21]
CCND1, CCND3, CDK18, CDK5, CDKN1A	Cell cycle	[22]
BRCA2, HLA-DQA1, MCM4, NOX1, PDCD6, PECAM1, PLD1, PPIC, RHOBTB2, SEC13, SOS1, VCAN, XPA	Immune response, lipid metabolism, cell cycle, glucose metabolism, DNA repair, Rho signaling, platelet signaling, insulin receptor signaling	[23]
ADCY9, TNNI3, RAPGEF3, CACNA1S, CACNG3, ADRA1B, CAMK2D, PPP2R3C, PPP2CA	Adrenergic signaling in cardiomyocytes	[24]
MFSD1, ARHGEF1	Immune response, positive regulation of secretion, regulation of blood circle, actin-mediated cell contraction, carbon metabolism, propanoate metabolism, 2-oxocarboxylic acid metabolism, biosynthesis of amino acids, prostate cancer pathways	[26]
HOXA5, ITLN1, RARRES3, RSPO3, HOXC10, CLDN1, RARRES1, KRT19, SLPI, CFI, IL18, MEIS1, TFPI2	HIF-1-, Toll-like receptor-, cGMP-PKG-, NF-kappa B-, p53-, AMPK-, Ras-, Hedgehog-, PI3K-Akt-, Wnt-, Hippo-, Rap1-, IL-17-, prolactin- signalling pathways	[27]
TGFB1I1, VNN1, HLADRB4, CXCL8, FN1, BPTF, PDE3B;	Granulocyte adhesion and diapedesis, pathogenesis of multiple sclerosis, macropinocytosis signaling, HER-2 signaling in breast cancer, B cell development, immune response	[28]
MEF2C, CDC42SE2	Muscular, vascular, neural, megakaryocyte, platelet development, B-cell signaling, immune response, regulation of actin cytoskeleton, phagocytosis	[29]
HNRNPUL2, HLA-DRB1, CXCL9, NPAP1L, HNRNPUL1, HLA-DRB5, FAM96A, NEB, SCXA, SPATA6	Immunological disease, cell morphology, immune response, cell cycle, PI3K/AKT signaling, NK-kB activation, PPAR signaling, cancer signaling,	[30]
ACTN1, AKT3, ARF6, ARHGAP6, ARHGEF2, CDH15, CDH6, CTNN, DLC1, GNA11, GNAL, GNAZ, GNB5, GNG11, ITGA2B	Cancer signaling, cellular movement and development, hematological system development and function, gene expression, cell death and survival, immune response, cell-to-cell signaling and interactions, connective tissue disorders, cell morphology, cellular function and maintenance	[31]

FOXO3, BCL2, CYBB, IL6, G6PC2, PHKA1, PTPN1, RPS6KB1, SORBS1, CHEK1, CDK1, CDC14A, STAG1, TFDP1, TFDP2	Cell cycle, cancer signaling, cellular senescence, focal adhesion, HIF-1 signaling, oxidative phosphorilation, infectious disease, regulation of actin cytoskeleton, cytokine-cytokine receptor interaction, insulin signaling	[32]
UBN1	Endoplasmic reticulum protein localization, translational elongation, generation of precursor metabolites, protein polyubiquitination	[33]
CL20, CXCL9, CXCL10, CXCL11, CXCL13, CCL18, JAK3, STAT1, STAT2, TNFSF10, TNFSF15, OAS1, OAS2, OAS3, IFIH1	Immune response, JAK/STAT signaling, TNF superfamily signaling, infectious disease, cell adhesion molecules pathway	[35]
UTY, FERP1, GCNT3, SAMD11, PKD2L2, CRLF1, SLC26A5, WDR38, C3orf65, MT-ND1, SLC12A1, MT-ND2, CACNA1H, MT-ND4L, EGFL8,	Immune responce, actin cytoskeleton, chromatin signaling	[37]
SLC12A1, THBD, TUBA1B, TUBA1A, PROK2, LILRA3, NFE2, AHNAK, HLX, ZNF641, CXCR1, GPR27, GPR27, TNFSF10, TLR1	Myeloid leukocyte activation, T cell activation, adaptive immunity, cytokine production, cytokine-cytokine receptor interaction pathway, NF-κB signaling, cell adhesion molecules, chemokine signaling	[38]
MMP8, MMP7, CCL18, SPP1, RNF128, CXCL10, RPS4Y1, LBP, AGXT2L1, CH25H, IL1RN, ESM1, PHACTR3, LIPN, TULP2	Fatty acid synthesis, mitochondrial function, innate immunity, transcriptional regulation	[39]
AKT2, DCL6, LIPE, METTL9, CD59, LMNA, FOS, CCL2, MUSTN1, ZNF638	Cholesterol metabolism, glycose metabolism, PPAR signaling pathway, maturity onset diabetes of the young, fatty acid degradation	[40]
SLC9A4, NECTIN2, PLPP3, LILRB5, AKR1C1	Immune response, lipid metabolism	[41]
INS, FXYD2, GPD2, LEPROTL1, RGS4, WFS1	Energy metabolism in mitochondria, protein synthesis, apoptosis, cytokine signaling	[42]
RASGRP1, GLP1R, GLRA1, LRFN2, ATP2A3, IL7R, SFRP4, CHL1, FAM105A, CLMP, KIAA1199, PFKFB2, ABCC8, FGF7, IL1R2	Islet cells growth, non-islet cells growth and proliferation	[43]
INS, STX1A, DLK1, GDA, CD36, RCOR1, LAPTM4B	Insulin signaling, immune response, nucleotide metabolism, intracellular signaling, chromatin regulation	[45]

The table shows up to 15 significant DEGs from each study (sorted based on *p*-value) and enriched pathways (if given) or biological functions of the genes.
